# Alpha-ketoglutarate for adipose tissue rejuvenation

**DOI:** 10.18632/aging.103853

**Published:** 2020-07-29

**Authors:** Qiyu Tian, Xiangdong Liu, Min Du

**Affiliations:** 1 Nutrigenomics and Growth Biology Group, Department of Animal Sciences, Washington State University, Pullman, WA 99164, USA

**Keywords:** alpha-ketoglutarate, aging, DNA methylation, stem cells, brown adipose tissue

Alpha-ketoglutarate (aKG), also called 2-oxoglutarate, is an intermediate of the TCA cycle in mitochondria. The mitochondrial membrane is highly permeable to aKG, which is mediated by malate/aKG and aspartate/glutamate exchangers located on mitochondrial membrane, which supplies aKG in the cytoplasm. In addition, aKG is also produced through oxidative deamination of glutamate and glutamine in cytosol. As a key metabolite, aKG is required for catalytic reaction of ten eleven translocation (TET) family of proteins, and TETs convert 5-methyl-cytosine (5mC) to 5-hydroxy-methyl-cytosine (5hmC), a key step in active DNA demethylation in an aKG-dependent manner [1]. In addition, aKG is also required for histone demethylation mediated by JmjC domain-containing histone demethylases, which catalyze the removal of repressive histone methylation marks (H3K9me3 and H3K27me3) and activate the expression of key developmental genes during stem cell differentiation [2].

Stem/progenitor cells generate new cells to replace senescent cells and maintain tissue homeostasis. However, stem/progenitor cells contain small amount of mitochondria and cytosol, which only contain very limited amount of aKG in contrast to its high demand for catalyzing DNA demethylation and other reactions, making aKG a rate limiting factor for TET-catalyzed DNA demethylation during stem/progenitor cell differentiation [3]. The aKG deficiency in stem and progenitor cells is worsened due to aging. As animals become old, mitochondrial function is progressively impaired and the cellular metabolic flux in the mitochondria declines, exacerbating aKG deficiency. Consistently, aberrant DNA methylation increases during aging [4]. The aKG deficiency associated with aging hampers DNA methylation or histone methylation which is required for stem/progenitor cell differentiation, suppressing their differentiation capacity and tissue homeostasis during aging. Indeed, compared to young mice, we found the aKG concentration is lower in aged mice [5].

Since the identification of abundant metabolically active BAT in adult humans in 2009 [6], the central role of brown and beige fat in obesity and metabolic dysfunction prevention has been rapidly recognized. BAT mass is negatively correlated with obesity in humans. However, during aging, the ability to form new brown/beige adipocytes declines and brown-to white transitioning is observed in subcutaneous WAT [7]. The PRDM16 is a key transcription factor governing brown adipogenesis and we found that aKG is a rate-limiting factor for the DNA demethylation of *Prdm16* and the consequent brown adipose tissue development [3]. Therefore, elevation of intracellular aKG level is postulated to rejuvenate the ability of progenitor cells to form new brown/beige adipocytes in aged mice. As expected, we demonstrated that dietary aKG supplementation replenishes the aKG pool of aged mice, which enhances brown and beige adipocyte formation and improves metabolic health in mid-aged mice [5].

Aging is not only associated with increase in metabolic diseases, but also cancer development. Carcinogenesis is associated with gaining of stemness and loss of differentiation capacity. In support, isocitrate dehydrogenase 1 (IDH1) and IDH2, two key enzymes catalyzing the formation of aKG, are frequently mutated in cancer cells, resulting in the generation of 2-hydroxyglutarate (2HG) instead of aKG [2]. The 2HG is a reduced product of aKG, which competitively inhibits aKG-dependent dioxygenases, blocking progenitor differentiation and promotes carcinogenesis. Consistently, aberrant DNA methylation in CpG has been commonly observed in tumors.

In our study, we supplemented middle-aged mice with aKG and found that dietary supplementation of aKG elevates its circulatory level and the availability of AKG for beige adipogenesis. As a result, DNA demethylation in *Prdm16* promoter and brown/beige adipogenesis was enhanced in aged mice [5]. Moreover, aKG supplementation improved metabolic health in aged mice challenged with high fat diet. In this study, we used 1% of aKG in drinking water for supplementation, which is relevant to aKG intake in humans. As a major metabolite and a derivative of dietary glutamine, a major amino acid, the aKG contents in foods are relatively high. In a study using growing pigs, the portal appearance of absorbed dietary aKG was limited and about 80% aKG disappears from the lumen of the small intestine due to its utilization by epithelial and splanchnic tissues [8].

In summary, through affecting histone and DNA demethylation, aKG is a critical mediator of cell differentiation and tissue homeostasis. While its level declines during aging, dietary supplementation of AKG increases intracellular aKG level, which can rejuvenate stem/progenitor cell function during aging, maintaining the health of adipose tissue. Because active DNA demethylation is required for the differentiation of progenitor cells in general, the dietary aKG intervention might prevent the senescence of other tissues due to aging as well.
